# Quality of Life and Disability Among Migraine Patients: A Single-Center Study in AlAhsa, Saudi Arabia

**DOI:** 10.7759/cureus.19210

**Published:** 2021-11-02

**Authors:** Hussain A Al Ghadeer, Sadiq A AlSalman, Fatimah M Albaqshi, Safaa R Alsuliman, Fatimah A Alsowailem, Haidar A Albusror, Zainab I AlAbdi, Esraa M Alwabari, Zainab A Alturaifi, Ahmed M AlHajji

**Affiliations:** 1 Paediatrics, Maternity and Children Hospital, AlAhsa, SAU; 2 Neurology, King Fahad Hospital-Hofuf, AlAhsa, SAU; 3 Neurology, King Faisal University, AlAhsa, SAU

**Keywords:** saudi arabia, migraine related disability, quality of life, chronic disease, headache, migraine

## Abstract

Background

Migraine is a major public health issue that leads to frequent visits to medical care. It is generally considered a disabling disease among individuals below the age of 50 years old predominantly seen in females. Migraine headache has a strong influence on disability, functional impairments, and psychological effects. The majority of the physicians fail to address the degree and extent of impediment caused by a migraine, which contributes to low quality of life and disability. Thus, this study aims to assess the health-related quality of life (QOL) and disability among migraine sufferers in AlAhsa, Saudi Arabia.

Material and methods

This descriptive cross-sectional study was carried out in the neurology clinics at King Fahad Hospital-Hofuf, AlAhsa, Saudi Arabia, from May to August 2021. The data were collected through a self-administered questionnaire. The migraine-specific quality of life questionnaire (MSQ), version 2.1, was used. MSQ is measured in three domains, including role function restrictive (RR), preventive (RP), and emotional function (EF). Disability related to migraine was assessed by the Migraine Disability Assessment Test (MIDAS). MIDAS classifies disability from no disability to severe disability. Two-tailed with an alpha level of 0.05 considering the significance of a p-value less than or equal to 0.05. The mean scores of QOL domains were compared by one-way analysis of variance (ANOVA) and independent t-tests.

Results

A total of 101 out of 359 participants were identified to have a migraine. Eighty-two participants met the inclusion criteria, 75.6% were females. The age of the participants ranged from 18 to more than 45 years with a mean age of 36.4 ± 11.9 years old. The mean score of QOL in the restrictive, preventive, and emotional domains were 46.3% ± 21.5%, 52.1% ± 24.3%, and 61.5% ± 30.8%, respectively. More than half of the participants (57.3%) suffered from a severe disability caused by migraines as compared to 20.7% with a moderate disability. Low QOL scores were associated with females and a significant relationship was found between migraine-associated disability and patients’ emotional function in QOL.

Conclusion

Clinicians should routinely evaluate disability related to migraine and QOL as a complementary approach to migraine patients to ensure that patients are receiving proper treatment and whether additional strategies are needed or not.

## Introduction

Migraine is a debilitating neurological condition that presents with moderate to severe headaches, most of the time reported to be unilateral and throbbing. It is often accompanied by sound sensitivity, light sensitivity, nausea, and vomiting. According to the International Headache Society's 3rd edition of the International Classification of Headache Disorders, headaches can be classified into primary and secondary headaches. Primary headaches include migraine, tension-type headaches, trigeminal autonomic cephalgia, and other primary headache disorders that do not belong to the other three groups and do not have a secondary cause. Secondary headaches can be caused by a wide spectrum of medical conditions that should be excluded before making the diagnosis of a primary headache. Migraine is a terrible headache characterized by pulsing headaches that last from a few hours to several days and are accompanied by nausea, vomiting, and/or photophobia and phonophobia. It can also be aggravated by physical exercise and tends to interfere with it. In 65%-70% of cases, migraine is unilateral, but it can be bilateral especially in children [1]. A cross-sectional survey published in 2020 found that the prevalence of migraine headaches in Saudi Arabia is higher compared with the global average and is associated with the female gender. The prevalence of all types of headaches in Saudi Arabia is estimated to be 77.2%, and migraine headache prevalence is around 25%. tension-type headaches (TTH) and migraines are the most common types of headaches. They are the second and third most common diseases in several versions of the Global Burden of Disease (GBD) survey [2]. Other researchers found that 14% of the population have migraine worldwide. Migraine is a major cause of disability [3]. A study has been done globally considering migraine the eighth disease associated with disability for many years, showing its burden [4]. Migraine headache impacts the quality of life causing missed days of school or work, reducing productivity, and causing a financial burden with the cost of the medications [5]. The quality of life in migraine patients was found to be significantly affected, causing major disability [6]. With frequent attacks, patients were found to be affected psychologically, socially, academically, and occupationally. The recurrent attacks cause functional impairments, which can involve both physical and psychological effects, which might happen during or after a migraine attack [7-10]. Comparing migraine sufferers and non-migraine sufferers, migraine sufferers have worse subjective well-being and lower quality of life [10]. Migraine is strongly associated with many psychiatric disorders, involving depression, anxiety, and bipolar disorders [11]. Studies found that migraine headache has a strong relationship with both depression and anxiety. Patients with both major depression and an anxiety disorder are at a two-fold higher risk to develop migraines compared with non-affected people. And people who have migraines are at a 2.2 to 4.0 times higher risk to develop depression [12-13].

## Materials and methods

Aim

This study aims to explore the health-related quality of life and disability associated with migraine headaches in AlAhsa, Saudi Arabia

Study design and participants

This cross-sectional study was conducted in neurology clinics at King Fahad Hospital-Hofuf, AlAhsa, in the Eastern province of Saudi Arabia from May 2021 to August 2021. People who were diagnosed clinically with migraine headaches or showed to have migraine headaches through migraine screening at the age of 18 years or older were included in the study. Patients were excluded if they had recently been diagnosed with migraine and had comorbidities other than migraine.

Data collection instrument and procedures

The data were collected from migraine patients who are attending the neurology clinics at King Fahad Hospital-Hofuf, AlAhsa, Saudi Arabia. Informed consent was obtained from all the participants after describing the aim of the study, and the participants had autonomy for rejection. The privacy and confidentiality of results were maintained. Official permission was obtained from the Institutional Review Board (IRB) of King Fahad Hospital-Hofuf, AlAhsa, Eastern province of Saudi Arabia (NO: 31-42-2021). An online or paper-based questionnaire was distributed. The questionnaire was composed of four parts: the first part was about biographical data, including age, gender, marital status, education level, household income, and several questions for screening of migraine headaches. The second part was to assess the social and work limitations in migraine patients. The third part measured the impact of headaches and determines the level of pain and disability caused by headaches using the five-item questionnaire designed to evaluate disability within the most recent three months. We used three valid and reliable questionnaires: the migraine-specific quality of life questionnaire and the migraine disability assessment test.

Migraine-specific quality-of-life questionnaire

The migraine-specific quality of life questionnaire measures the effect of headaches on the patient's life in the past four weeks. It contains three domains: role function-restrictive (RR), role function-preventive (RP), and emotional function (EF). A 14-item questionnaire was used to assess daily social and work limitations, and evaluate the emotion associated with migraines. All domains of MCQ are scaled from 0 to 100; higher scales demonstrate better functioning. Items contain six choices, ranging from none of the time to all the time, with standard six points ordered on a categorical scale. Many studies test the validity and reliability within different languages, including the Arabic version and it was appropriate [14-15].

Migraine disability assessment test

The purpose of the migraine disability assessment test (MIDAS) questionnaire is to quantify the disability related to migraine for three months. The questionnaire determines the level of pain and disability. It aims to measure the impact of migraines on life and involves five questions in three dimensions of activity. The first and second questions are related to the school/work dimension. The third and fourth questions are related to the housework dimension. The last questions are related to the social dimension. There are two questions associated with clinical value. The first question is: How many days in the last three months did you have a headache? The second question is: On a scale of 0-10, on average, how painful were these headaches? The four MIDAS grades are associated with the MIDAS score. Grade I is from the 0 to 5 score, which indicates little or no disability. Grade II is from the 6 to 10 score, which indicates mild disability. Grade III is from the 11 to 20 score, which indicates moderate disability. Grade IV includes scores above 20, which indicates severe disability [16-17].

Data analysis

The data were collected, reviewed, and then fed to the Statistical Package for Social Sciences version 21 (IBM Corp., Armonk, NY). All statistical methods used were two-tailed with an alpha level of 0.05 considering significance at a p-value of less than or equal to 0.05. In migraine-specific quality-of-life domains (role function restrictive, role function preventive, and emotional function), the standardized scores were calculated with reference to the questionnaire-specific transformation formula, to have a standardized score of 100 for each domain. Regarding MIDAS, the total number of days was calculated and categorized with reference to the scale cut-off points. Descriptive analysis using frequency and percent distribution for all categorical variables was applied including disability level. Mean with standard deviation was used to describe the distribution of migraine-specific quality of life domains. Cross-tabulation for showing the distribution of participants’ disability level according to their bio-demographic data was carried out with Pearson chi-square test for significance and exact probability test for small frequency distributions. One-way analysis of variance (ANOVA) and independent t-tests were used to compare the mean scores of QOL domains by participants' personal data.

## Results

Eighty-two participants fulfilled the inclusion criteria, with clinically diagnosed migraine and having completed the study questionnaire. The participant's ages ranged from 18 to more than 45 years with a mean age of 36.4 ± 11.9 years old. An exact of 62 participants (75.6%) were females. Single participants were 15 (18.3%) and 63 (76.8%) were married. Considering educational level, 55 (67.1%) participants were university graduates and 19 (23.2%) had a below-university level of education. An exact of 44 (53.7%) participants were not employed, 26 (31.7%) were non-healthcare workers or students, while 12 (14.6%) were health care, workers/students. Monthly income less than 5000 SR was reported among 20 (24.4%) participants, and 30 (36.6%) had a monthly income of 5000 to 10000 SR (Table 1).

**Table 1:** Sociodemographic data of study participants with migraine in AlAhsa, Saudi Arabia

Sociodemographic data	Count	Column N %
Age in years		
< 25	15	18.3%
25-35	29	35.4%
36-45	27	32.9%
> 45	11	13.4%
Gender		
Male	20	24.4%
Female	62	75.6%
Marital status		
Single	15	18.3%
Married	63	76.8%
Divorced / widow	4	4.9%
Educational level		
Below university	19	23.2%
University	55	67.1%
Post graduate	8	9.8%
Job title		
Not employed	44	53.7%
Non health care worker / student	26	31.7%
Health care worker / student	12	14.6%
Monthly income		
< 5000 SR	20	24.4%
5000-10000 SR	30	36.6%
10000-20000 SR	24	29.3%
> 20000 SR	8	9.8%

Table 2 lists the drugs and methods used by study participants to relieve migraine attacks, AlAhsa, Saudi Arabia. An exact of 61 (74.4%) participants reported using drugs to relieve migraine attacks. The attack severity was relieved by less than 25% of its severity among 33 (40.2%) of those who had drugs, but 23 (28%) reported relief of 25-50%, and only 12 (14.6%) were relieved by more than 75%. Considering alternative methods, 30 (36.6%) reported using Islamic therapy (Ruqia, Quran), 23 (28%) used traditional medicine (herbs), and 22 (26.8%) used Cupping therapy, while 23 (28%) used none of them. Among users, attack severity was relieved by less than 25% among 46 (56.1%) while nine (11%) reported pain relief exceeding 75%. Smoking was reported by 10 (12.2%) participants while 79 (96.3%) had coffee and tea but soft drinks were taken by 28 (34.1%).

**Table 2:** Drugs and methods used by study participants to relieve migraine attack in AlAhsa, Saudi Arabia

Methods	No	%
Had drugs to relieve migraine attacks		
Yes	61	74.4%
No	21	25.6%
Degree of improvement in migraine attacks after having treatments		
< 25%	33	40.2%
25-<50%	23	28.0%
50-75%	14	17.1%
> 75%	12	14.6%
Alternative medicine usage		
Traditional medicine (herbs)	23	28.0%
Chinese acupuncture	3	3.7%
Cupping therapy	22	26.8%
Islamic therapy (Ruqia, Quran)	30	36.6%
Others	12	14.6%
None	23	28.0%
Degree of improvement in migraine attacks after these methods		
< 25%	46	56.1%
25-<50%	13	15.9%
50-75%	14	17.1%
> 75%	9	11.0%
Usage any of the following frequently		
Smoking	10	12.2%
Power & soft drinks	28	34.1%
Tea & coffee	79	96.3%

**Figure 1:**
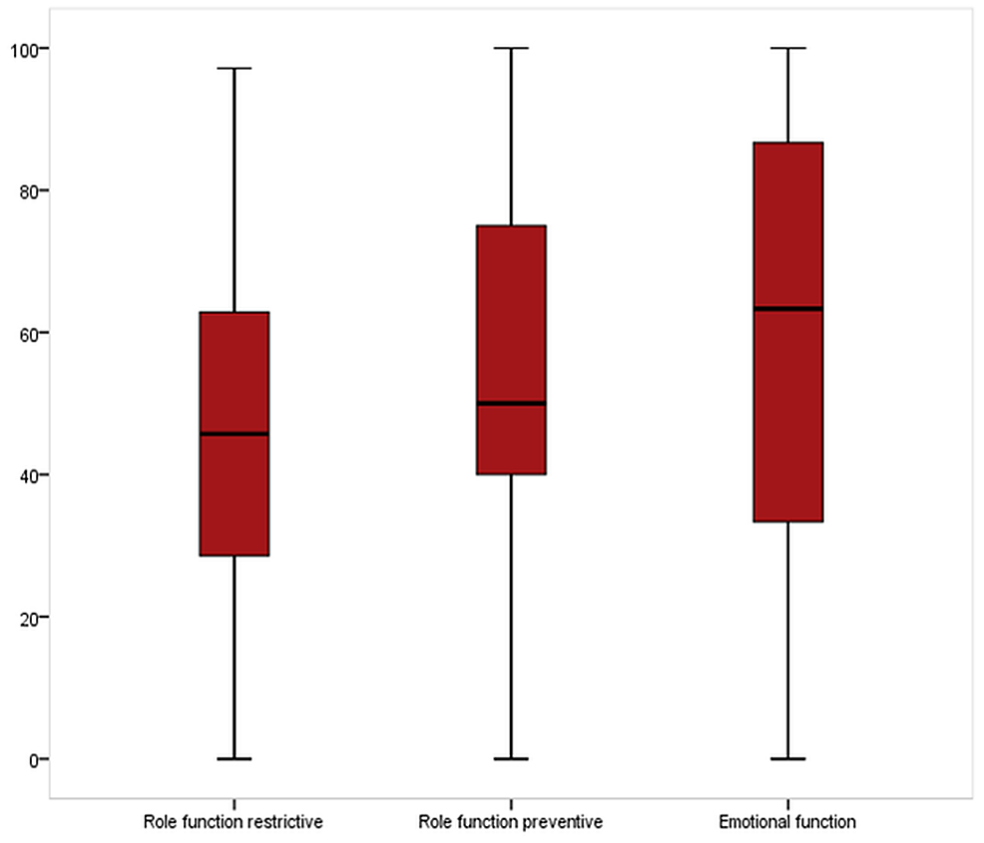
Box-plot showing the distribution of migraine-specific quality-of-life domain score among the population pf Al-Ahsa, Saudi Arabia

Figure 1 presents a box-plot showing the distribution of migraine-specific quality-of-life domain scores among the population of AlAhsa, Saudi Arabia. For the role function restrictive domain, the participant's score ranged from 0% to 97% with a mean score of 46.3% ± 21.5%. Considering the role function preventive domain, participants’ scores ranged from 0% to 100% with a mean score of 52.1% ± 24.3%. About the emotional function domain, participants’ core ranged from 0% to 100% with a mean score of 61.5% ± 30.8%.

Table 3 shows the distribution of QOL domains among participants with a migraine, AlAhsa, Saudi Arabia. The role function restrictive domain was significantly higher among females (50.7) than males (32.7) and among less-educated persons (39.8) than participants with a postgraduate degree (31.1). Also, this domain score is significantly higher among non-employed than others. The mean score was significantly higher among those who did not have drugs to relieve pain than others who did (58.9 vs. 42.1). Considering the role function preventive domain, its score was significantly higher among females than males (56.6 vs. 37.7), among non-employed than employed persons, (57.4 vs. 36.3), and among drugs non-users than users (65 vs. 47.5). Regarding emotional function scores, it was significantly higher among drugs nonusers than among users (74.9 vs. 586.8) and among those who did not use alternative methods than others who use (74.5 vs. 56.4).

**Table 3:** Distribution of QOL domains among participants with migraine in AlAhsa, Saudi Arabia P: one-way analysis of variance (ANOVA); $: independent t-test; * P <0.05 (significant)

Factors	Role function restrictive		Role function preventive		Emotional function	
	Mean	SD	Mean	SD	Mean	SD
Age in years						
< 25	41.14	15.19	48.67	20.91	49.78	32.06
25-35	47.59	25.36	52.07	27.86	69.66	32.88
36-45	45.19	18.77	54.44	22.46	58.02	28.57
> 45	52.99	24.59	50.45	25.34	64.24	25.35
p-value	.559		.900		.201	
Gender						
Male	32.71	19.59	37.75	22.15	53.00	31.38
Female	50.74	20.33	56.61	23.27	64.19	30.33
p-value^$^	.001*		.002*		.159	
Marital status						
Single	45.33	15.90	49.00	23.24	63.56	24.80
Married	47.30	22.65	53.17	25.23	61.27	32.63
Divorced / widow	35.00	22.18	45.00	10.00	56.67	25.24
p-value	.535		.707		.921	
Educational level						
Below university	39.85	19.51	45.26	21.89	54.74	29.61
University	50.81	21.50	56.09	24.47	65.45	31.22
Postgraduate	31.07	16.27	40.00	23.45	50.00	28.06
p-value	.015*		.082		.232	
Job title						
Not employed	50.00	20.08	57.39	21.47	64.09	31.64
Non-health care worker / student	46.92	22.73	50.19	25.12	62.05	26.98
Health care worker / student	31.67	19.05	36.25	26.64	50.56	35.33
p-value	.030*		.023*		.404	
Monthly income						
< 5000 SR	48.29	27.17	57.50	26.28	71.00	36.21
5000-10000 SR	48.10	21.06	51.83	24.48	56.44	30.41
10000-20000 SR	45.71	17.73	52.29	21.87	61.67	28.44
> 20000 SR	36.79	18.48	38.13	24.19	55.83	22.52
p-value	.588		.306		.399	
Had drugs to relieve migraine attacks						
Yes	42.01	21.25	47.54	23.92	56.83	31.00
No	58.91	17.13	65.00	20.80	74.92	26.41
p-value^$^	.001*		.004*		.019*	
Degree of improvement in migraine attacks after having treatments						
< 25%	50.82	22.28	54.09	24.41	63.84	28.63
25-<50%	44.84	22.83	50.87	25.92	56.81	27.55
50-75%	44.90	12.14	52.86	15.28	65.24	36.63
> 75%	38.57	24.65	47.50	30.79	59.44	37.44
p-value	.370		.872		.811	
Alternative medicine usage						
Yes	44.55	21.47	49.15	24.60	56.38	30.08
No	50.93	21.34	59.35	22.27	74.49	29.24
p-value^$^	.229		.088		.016*	
Degree of improvement in migraine attacks after these methods						
< 25%	47.02	20.13	53.26	24.04	62.75	28.82
25-<50%	41.10	22.48	41.54	19.94	57.95	30.23
50-75%	47.55	19.37	53.21	21.00	57.14	39.20
> 75%	48.57	31.23	58.89	34.08	66.67	30.91
p-value	.817		.353		.858	

**Figure 2:**
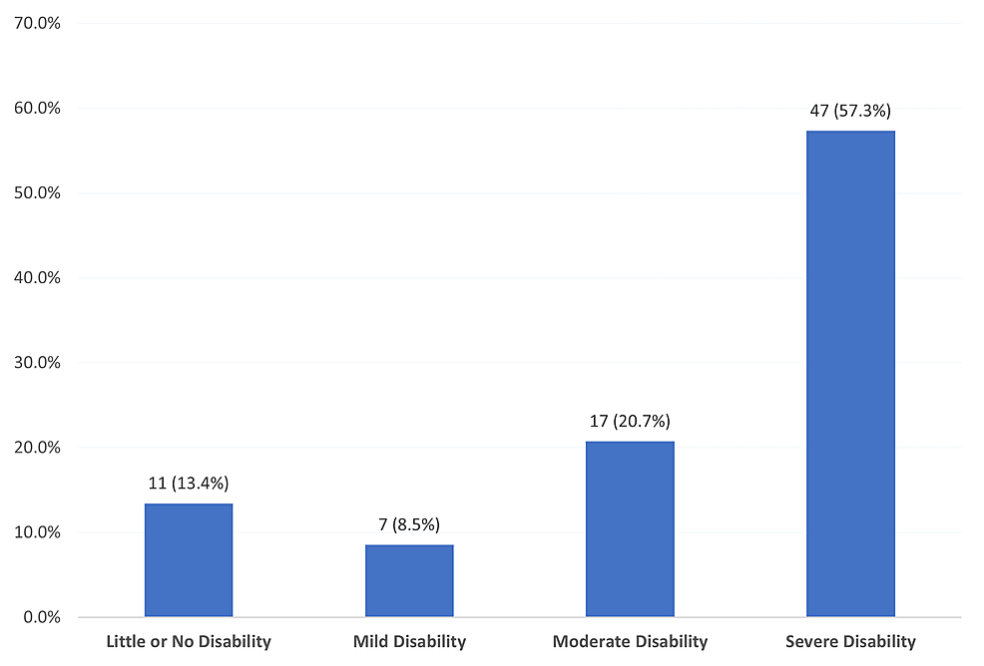
Degree of migraine-associated disability among study participants in AlAhsa, Saudi Arabia

Figure 2 shows the degree of migraine-associated disability among study participants in AlAhsa, Saudi Arabia. An exact of 11 (3.4%) had little or no disability, seven (8.5%) had mild disability, 17 (20.7%) had moderate disability, and 47 (57.3%) had severe disability.

Table 4 shows the distribution of migraine-associated disability by participants’ sociodemographic data. Moderate to severe migraine-associated disability was detected among 82.3% of females in comparison to 65% of males with recorded statistical significance (P=.048). Also, all health care workers/students had moderate to severe disability due to migraine compared to 46.2% of non-healthcare workers (P=.001). Moderate to severe disability was among 96.7% of participants with a monthly income of 5000-10000 SR versus 50% of those with more than 20000 SR (P=.011).

**Table 4:** Distribution of migraine-associated disability by participants’ sociodemographic data P: exact probability test; * P < 0.05 (significant)

Factors	Migraine-associated disability				p-value
	Little / mild		Moderate / severe		
	No	%	No	%	
Age in years					.465
< 25	1	6.7%	14	93.3%	
25-35	7	24.1%	22	75.9%	
36-45	7	25.9%	20	74.1%	
> 45	3	27.3%	8	72.7%	
Gender					.048*
Male	7	35.0%	13	65.0%	
Female	11	17.7%	51	82.3%	
Marital status					.380
Single	3	20.0%	12	80.0%	
Married	13	20.6%	50	79.4%	
Divorced / widow	2	50.0%	2	50.0%	
Educational level					.263
Below university	2	10.5%	17	89.5%	
University	13	23.6%	42	76.4%	
Postgraduate	3	37.5%	5	62.5%	
Job title					.001*
Not employed	4	9.1%	40	90.9%	
Non health care worker / student	14	53.8%	12	46.2%	
Health care worker / student	0	0.0%	12	100.0%	
Monthly income					.011*
< 5000 SR	6	30.0%	14	70.0%	
5000-10000 SR	1	3.3%	29	96.7%	
10000-20000 SR	7	29.2%	17	70.8%	
> 20000 SR	4	50.0%	4	50.0%	
Had drugs to relief migraine attacks					.325
Yes	15	24.6%	46	75.4%	
No	3	14.3%	18	85.7%	
Alternative medicine usage					.977
Yes	13	22.0%	46	78.0%	
No	5	21.7%	18	78.3%	

Table 5 shows the association between participants with migraine quality of life and migraine-associated disability. There was a significant association between migraine-associated disability and patient's emotional function score where the mean emotional function score was 75.2 ± 21.5 among participants with little/mild disability compared to 57.6 ± 32.0 among others with moderate/severe disability (P=.031).

**Table 5:** Association between participants with migraine quality of life and migraine-associated disability P: independent t-test; * P < 0.05 (significant)

QOL	Migraine-associated disability				p-value
	Little / mild		Moderate / severe		
	Mean	SD	Mean	SD	
Role function restrictive	49.2	21.3	45.5	21.7	.525
Role function preventive	56.7	25.1	50.7	24.1	.360
Emotional function	75.2	21.5	57.6	32.0	.031*

## Discussion

The current study was conducted to assess the quality-of-life impacts related to migraine headaches in AlAhsa, Saudi Arabia. It is also set to assess the disabilities related to migraine headaches. The incidence and severity of migraine attacks may increase over time to chronic migraine, as attacks tend to occur for at least 15 days in a month [18]. The persistent attacks may be a leading cause of considerable functional impairments [10], including physical and psychological disabilities [7] and impact on academic [8], work-related [9], social, and quality of life [10,17]. Migraine-associated impairments were reported throughout or between the migraine attacks. Commonly, patients with migraines had poor well-being and low quality of life during and in between the attacks than do age- and sex-matched healthy controls [10].

Regarding the migraine-related quality of life, the study revealed that the role function restrictive domain was below half of the maximum score (46%) while the highest score was for the emotional function domain where participants’ score was 61.5%, but for the role function preventive domain, the participants’ score was 52.1%. This implies that migraineurs had an average level of quality of life, especially in their role functions. The surprising findings were that higher quality-of-life scores were among less-educated persons and those who never used drugs or other alternative methods to relieve migraine-associated pain. This may be explained by two things. First, those who did not have any drugs or alternative methods to relieve pain may have minimal pain level, which explains the non-use of the drugs and so their quality of life was not highly affected in contrast to others who had drugs due to severe pain attacks, which highly influenced their social and physical life. Second, it was observed that the relief in pain severity with drug use was not high (mostly < 50%), which means still having pain among drug users affected their quality of life. Also, findings revealed that quality of life was better among male participants than females. These findings were consistent with what was reported by Shaik MM et al. [19], as the authors found that females with migraines had significantly lower total WHOQOL-BREF scores (84.3) than healthy controls (91.9, P<0.001). Likewise, physical health (23.4 versus 27.7, P<0.001) and psychological health scores (21.7 versus 23.2, P< 0.001) were significantly lower than those for healthy controls. Also, these findings show similar results to other studies, which estimated a lower QOL and health among migraineurs [20-21]. In the USA, a study was conducted among migraine patients, and the total QOL, physical health, and social functioning scores of the patients with migraine were considerably poorer than the published averages [22]. Another study among the Dutch population [23] revealed reduced functional ability and quality of life among migraineurs. Also, quality of life and well-being were significantly lower among UK [24], French [25], and Italian patients [26].

Regarding migraine-associated disability, the current study showed that more than half of the migraineurs had severe disability where only 13% had minimal or no disability. Disability was significantly higher among females, health care workers, and others with low-income levels. These findings are matching with what was reported by Pradeep R et al. [27] in which the majority of migraineurs had a highly significant disability that negatively impacted their quality of life in all the domains. There was also reported anxiety and depression with migraines. Park JW et al. reported that the average days noted with disability for one or more of the three aspects of activities per headache attack was 0.55 [28].

Regarding methods used to relieve migraine attack-associated pain, the current study showed that three out of every four participants with migraine used drugs with a reported low relief percentage (< 50%). Also, nearly one-third of them used herbs or Islamic therapy for relieving the pain besides drugs. Cupping therapy was used by one-quarter of the participants where many of them used more than one method. About half of those participants reported poor improvement in attack severity with the used methods.

## Conclusions

The current study established that there is a significant inverse effect of migraine-associated headache on migraineurs in all aspects of quality of life. Also, high disability was detected among persons with migraines especially health care workers and females. Generally, assessment of migraine headache must be comprehensive, including assessment of mental health, well-being, and real-life effect on the patient, therefore delivering effective management.
